# Antioxidant Potential of Natural Extract of *Ginkgo biloba* L. in Relation to Chorioallantoic Membrane (CAM) Vessels of Chicken Embryo

**DOI:** 10.3390/life15050827

**Published:** 2025-05-21

**Authors:** Miriam Bačkorová, Veronika Petruľová, Eva Petrovová

**Affiliations:** 1 Department of Pharmaceutical Technology, Pharmacognosy and Botany, University of Veterinary Medicine and Pharmacy, 041 81 Košice, Slovakia; veronika.petrulova@uvlf.sk; 2 Department of Morphological Disciplines, University of Veterinary Medicine and Pharmacy, 041 81 Košice, Slovakia; eva.petrovova@uvlf.sk

**Keywords:** blood vessels, CAM model, flavonoids, in vivo model, *Ginkgo biloba* L.

## Abstract

The chicken chorioallantoic membrane (CAM) model is an embryonic blood capillary system considered a suitable “in vivo” model for studying the irritation effect of plant extracts on the vascular system, including impacts on hemostasis, hyperemia, hemorrhage, and coagulation. The main aim of the present work was to investigate the irritation effects of different concentrated alcohol extracts of *Ginkgo biloba* L. (GBE) leaves on the blood vessels of the CAM model during early embryogenesis, evaluated using the Luepke scoring system. The antioxidant properties of GBE were assessed using DPPH radical scavenging and the FRAP method, alongside HPLC-DAD analysis to confirm the presence of major therapeutically relevant metabolites, revealing a strong therapeutic potential of the extract. On embryonic day 9, different concentrations of GBE as well as the controls (saline solution and 30% ethanol) were applied to the CAM surface. Vascular changes were observed immediately after application, with vasoconstriction leading to the temporary “disappearance” of blood vessels. At 30 s post-application, all GBE concentrations and ethanol induced hyperemia and mild hemorrhage, which gradually diminished over time. No changes were observed with saline application. The extent of morphometric changes in the vessels was also influenced by the concentration of GBE used. Concentrations of 20% and 30% GBE induced vasoconstriction. Lower concentrations of GBE induced vasodilation, with maximum values recorded after 240 s for 1% and 15% GBE. The results of this study may help to better characterize the vascular effects of natural *Ginkgo biloba* under in vivo conditions and promote greater interest in the use of alternative animal models in pharmacological and biomedical research.

## 1. Introduction

*Ginkgo biloba* (GB) is the oldest deciduous dioecious tree in the *Ginkgoaceae* family. Historically, it dates to 170 million years ago and geologically to the Jurassic period. For this reason, it also has the name “living fossil” of use in traditional Chinese medicine [1]. The main components of GB extract include flavonoids, terpenoids (ginkgolides and bilobalide), polyprenols, polysaccharides, and organic acids [2]. Flavonoids are the most important secondary metabolites in GB leaves that determine their medicinal quality. It was found that flavonoids in the human body increase the resistance of capillaries by increasing their flexibility and preventing their permeability. In addition, they are also involved in the stimulation of heart activity; at the same time, they have diuretic and antimicrobial properties. Flavonoids in the aging process improve the function of mitochondrial respiration. They protect the brain by absorbing neurotransmitters, inhibiting apoptosis in sensory tissues. This process takes place in the olfactory bulb and in the pigment epithelium of the retina of the eye [3,4]. Leaf extracts of GB (GBEs) are used to treat dementia complications such as difficulty concentrating and memory impairment. These extracts also have antiasthmatic, antioxidant, wound healing, and reactive oxygen species (ROS) protection properties, as well as neuroprotective properties against neurodegenerative disorders such as Alzheimer’s disease and Parkinson’s disease [5,6].

The chorioallantoic membrane (CAM) of the avian embryo is a structurally simple, highly vascularized extraembryonic membrane. It is formed on embryonic day 4 by the fusion of the chorion and allantois. After the onset of development of the avian embryo, it begins to function. In addition to the respiratory function, which involves the transport of gases through the pores of the shell, the CAM also provides an excretory function [7,8]. Another function is transport from the shell, due to bone mineralization and the transport of sodium and chloride [9]. In addition to the vascular system, the CAM has a fully developed lymphatic system that is very similar to that of mammals [10].

Histologically, the CAM consists of two epithelial layers that line a thin stromal layer of tissue [11]. The upper epithelial layer, which originates from the chorion, is of ectodermal origin, whereas the stromal layer and the lower epithelial component are of mesodermal and endodermal origin. The functional stromal components of the CAM are blood and lymphatic vessels. Regarding the structural elements of CAM, it is important to remember that the main topically applied component must pass through the outer epithelium and reach the vessels located in the stromal component. In ex ovo culture procedures, there is sufficient humidity. It reduces humidity to significantly increase cell division and keratinization of the outer single-layered (under normal circumstances) epithelium. The CAM epithelial layer is thicker, which limits the distribution of topically applied substances to the CAM vasculature. The relatively rich vascularized membrane of the avian embryo is also the membrane of the yolk sac. From a developmental point of view, it performs different functions than the CAM. In the early stages of embryo development, both membranes are easily distinguishable; in later stages, the differences are less obvious. In the early stages of embryo development, the attachment of the CAM to the inner membrane of the shell occurs, so it is very important to make the experimental area accessible before this happens [12]. Thanks to the mentioned properties, this experimental model is very suitable for the purposes of research aimed at the study of angiogenesis and related processes [13].

In recent years, research has focused on the search for new natural medicines as potential therapeutic agents. Natural compounds should have a chemopreventive effect with minimal toxic effect on normal, healthy cells. The endothelium of blood vessels is highly sensitive to elevated levels of ROS, which can arise due to vascular senescence, mitochondrial dysfunction, or chronic low-grade inflammation. Plant extracts help mitigate oxidative stress through various mechanisms, including direct quenching of ROS by hydroxyl groups in polyphenolic compounds (such as flavonoids, organic acids, or caffeoylquinic acid derivatives). Additionally, they enhance the activity of antioxidant enzymes like glutathione (GSH) and superoxide dismutase (SOD), modulate the expression of vasoprotective transcription factors, and reduce the expression of endothelial cyclooxygenase (COX) and NADPH oxidase (Nox), both of which are involved in ROS production [14].

Testing natural substances on the chorioallantoic membrane (CAM) of the avian embryo has been classified as a simple, rapid, and inexpensive model for investigating the effects of plant substances applied topically or injected intravascularly on amnion toxicity [15].

The main objective of this study was to monitor the irritation effect of GBE applied at different concentrations on the vessels of the chicken embryo chorioallantoic membrane and to evaluate it using the Luepke scoring system. To better understand the demonstrated effects of GBE, the antioxidant potential of GBE at different dilution levels and its metabolite composition were evaluated.

## 2. Materials and Methods

### 2.1. Preparation of Plant Extract

The leaves of GB were obtained from the specimens growing in the Botanical Garden of Pavol Jozef Šafárik University in Košice (48°44′06.9″ N 21°14′12.6″ E), in June 2019. The leaves were air-dried in a dark room to preserve their chemical composition. After drying, 10 g of leaf drug was ground to a fine powder and extracted with 200 mL of ethanol in an ultrasonic bath (KRAINTEK 18, Kraintek s.r.o., Podhájska, Slovakia) for 30 min, where the temperature did not exceed 40 °C. After the first extraction, the mixture was filtered, and the remaining plant residue, together with the filter paper, was re-extracted with ethanol to maximize the yield. This extraction and filtration process was repeated a total of three times. During extraction and filtration, all laboratory glassware was protected from light to prevent the degradation of sensitive compounds. The total extract was evaporated to dryness using a rotary vacuum evaporator (Heidolph Hei-Vap Precision, Schwabach, Germany). The extract contained 1.0 g of the drug in 1.0 mL of ethanol.

### 2.2. Sample Preparation for HPLC

Dry GB leaves (1 g) were extracted with 1 mL ethanol (100%, Sigma Aldrich, St. Louis, MO, USA) in four repetitions. After centrifugation (5000× *g*, 5 min), 100 µL of filtrated extract (syringe filter 0.22 µm, 5 mm) was diluted in 900 µL of ethanol. From that, 20 µL was used for the determination of flavonoid glycosides by HPLC-DAD (high-performance liquid chromatography) (Agilent Technologies, St. Louis, MO, USA). For the determination of flavonoid aglycons, acid hydrolysis was used. Here, 50 µL of 1M HCL was added to 450 µL of ethanolic extract, and the mixture was heated for 1 h at 90 °C. Then, 20 µL filtrated hydrolysate was used for HPLC-DAD analyses of aglycons. All HPLC-DAD measurements were conducted in eight repetitions. Purchased standards, such as quercetin (purity ≥ 95%, Sigma Aldrich, St. Louis, MO, USA), kaempferol (purity ≥ 95%, Roth), isorhamnetin (purity ≥ 95%, Roth, Germany), rutin (purity ≥ 95%, Sigma Aldrich, St. Louis, MO, USA), isoquercetin (purity ≥ 95%, Roth), kaempferol-*3*-glucoside (Extrasynthése, Genay, France), isorhamnetin-*3*-glucoside (Extrasynthése), and (+)-catechin (purity ≥ 98%, Sigma Aldrich, St. Louis, MO, USA), were used for quantitative and qualitative analyses. From the obtained chromatograms, the determination of flavonoid glycosides was carried out based on agreement in chosen features (retention times, absorption maxims, shape of UV spectral curves) using appropriate standards. Acid hydrolysis of GBE caused the release of aglycons, and their qualitative and quantitative determinations were carried out. For the construction of the calibration curves of the detected metabolites, four different volumes (5, 10, 15, and 20 µL) in three repetitions were taken from stored standard solutions.

### 2.3. Conditions of HPLC-DAD Analyses

An Agilent Technologies 1260 Infinity (Agilent, Santa Clara, CA, USA), consisting of a degasser, an autosampler, a binary pump, and a DAD detector, was used for chromatographic analyses. The separation of methanolic and hydrolysated extracts was carried out using a Kromasil 100 C18 (5 µm, 250 × 4.6 mm; Göteborg, Sweden), with mobile phases A (5% acetonitrile with 5% of trifluoracetic acid) and B (80% acetonitrile) in the following gradient program: 0 min (A 100%), 15 min (A 60%), 30 min (A 40%), 35 min (A 0%), 45 min (A 0%), and 50 min (A 100%), with a flow rate of 0.7 mL min^−1^. The wavelength chosen for the detection of flavonoids was 350 nm, and that for (+)-catechin was 220 nm.

#### Quantitative Determination of Flavonoid Content

An amount of 0.6 g DW was heated and extracted with a mixture of 0.5% methenamine, acetone, and concentrated hydrochloric acid (1/20/1 mL) for 30 min in a round-bottom flask in a water bath under reflux. After extraction, the mixture was cooled and filtered into a 100 mL volumetric flask. The extracted drug was again heated in the same flask in the same manner two more times with 20 mL of acetone for 10 min. Then, water (20 mL) and ethylacetate (15 mL) were added to 20 mL of the obtained extract, which was then placed in a separating funnel. For flavonoid determination, 1 mL of 2% AlCl_3_ (diluted in methanol with concentrated acetic acid in a ratio of 19:1) was added to 10 mL of the ethylacetate yield. After a 30 min reaction, the total flavonoids were spectroscopically detected at 425 nm [16].

### 2.4. Determination of Antioxidant Activity

The determination of GBE antioxidant activity was realized using DPPH and FRAP assays.

#### 2.4.1. DPPH Assay

Four different extract concentrations (20 mg/mL; 10 mg/mL; 5 mg/mL; 2.5 mg/mL) (2%, 1%, 0.5%, 0.25%) were prepared from the stored extract (described in Section 2.1.) and subjected to radical scavenging assays. The DPPH assay is based on the reaction of a purple-colored free radical with a scavenger to yield the colorless product 1.1-diphenyl-2-picrylhydrazine (Sigma Aldrich, St. Louis, MO, USA). For this, 100 µL of each diluted GBE was added to 900 µL of 0.2 mmol/L methanol DPPH solution. Incubation was carried out for 30 min at 25 °C. The negative control was a DPPH solution with 100 μL of methanol (Mikrochem, Pezinok, Slovakia). The decrease in absorbance was measured at a wavelength of 517 nm using a UV–Vis spectrophotometer (Beckman DU 530 UV/VIS, Brea, CA, USA). The DPPH assay was conducted in four repetitions for each GBE concentration. Antioxidant activity was expressed as the percentage decrease in radical AA (%), which was calculated as the decrease in the absorbance of the extracts (A) relative to the control (A_0_) [17]:

AA (%) = (A_0_ − A_x_)/A_0_ × 100

where A_0_ is the absorbance of the sample with DPPH, and A_x_ is the absorbance of the negative control.

#### 2.4.2. Ferric Reducing Antioxidant Power

The essence of the FRAP assay is the reduction of the colorless complex Fe^3+^-ferrictyridyltriazine (Fe^3+^-TPTZ or FRAP reagent) to the blue-colored complex Fe^2+^-tripyridyltriazine at low pH (3.6), using a 0.01 M solution of 2,4,6-tripyridyl-S-triazine (Sigma Aldrich, St. Louis, MO, USA) in 0.04 M HCl (Mikrochem, Pezinok, Slovakia) and 0.02 M ferric chloride (Mikrochem, Pezinok, Slovakia) in a ratio of 10:1:1 (FRAP solution). The preparation procedure, reagents, and evaluation of the determination followed the Aktumsek protocol [18]. Mixtures of GBE and FRAP reagent were spectrophotometrically detected after a four-minute reaction at a wavelength of 593 nm. FeSO_4_ at a concentration of 100 µmol/L was used as a standard, and antioxidant activity was expressed in FRAP units (1 FRAP = 100 µmol/L Fe^2+^). All the measurements were conducted in triplicate, and standard deviations were also provided.

### 2.5. CAM Model Preparation

The materials used in the method were a total of 60 hatching eggs of the Ross 308 breed, which were obtained from a hatching farm (Párovské Háje, Slovakia). Before incubation, the eggs were cleaned with 70% alcohol and placed in an incubator under standard conditions (37.5 ± 0.5 ° C, 60% humidity). On day 3 of incubation, 2 mL of protein was removed from each egg using a syringe and needle to minimize adhesion of the shell membrane to the chorioallantoic membrane of the chicken embryo (CAM). On embryonic day 9, a hole was cut at the blunt end of the egg, and the inner shell membrane was removed with tweezers. Subsequently, we applied various concentrations of GBE to the CAM (concentration of extract: 1%, 5%, 10%, 15%, 20%, and 30%). A physiological saline solution (0.9% NaCl) was used as a positive control, as it provides a neutral effect on blood vessels without any irritating properties. A 30% ethanol solution was used as a negative control. The changes were observed using an Olympus SZ61 (Olympus corporation, Tokyo, Japan) stereomicroscope with an ARTCAM-300MI digital camera (Tokyo, Japan). We followed the modified method of Luepke [19], in which the vasoactivity of the vessels of the chorioallantoic membrane is evaluated 30, 120, and 240 s after application of the test substance using Quick Photo 2.3 software (PROMICRA, Prague, Czech Republic). To minimize subjective observation, two independent researchers evaluated the irritant effects (4 eggs per solution in total). This method uses a numerical evaluation of vasoactivity, specifically hyperemia, hemorrhage, and coagulation, as a function of time (Table 1). The individual values for these changes are summed to give a single resulting numerical value, indicating the irritation potential of the test substance, on a scale with a maximum value of 21 (Table 2) (the value representing the greatest irritation). Also, we observed morphometric changes in vessel diameter [20] using the stereomicroscope images and evaluated them with QuickPHOTO MICRO 3.2 software (PROMICRA, Prague, Czech Republic). The blood vessel diameter was measured in 6 fields of view for each sample.

### 2.6. Statistical Analysis

One-way ANOVA was carried out using the statistical program GraphPad Prism 6.0, and *p*-values ˂ 0.05 were considered significant for the statistical evaluation of samples. Four eggs were used to assess each GBE concentration. At each concentration, a morphometric evaluation of vessel diameter was conducted on 12 to 18 vessels.

## 3. Results

The main objective of this study was to determinate the irritation effect of GBE applied at six concentrations on the chicken embryo chorioallantoic membrane evaluated using the Luepke scoring system. The determination of antioxidant activity and the presence and content of flavonoids can provide a better understanding of the effect of GBE on the CAM system.

### 3.1. Antioxidant Activity Using FRAP and DPPH Assays

The determination of the antioxidant activity of GBE was realized using the DPPH assay, which is widely used to evaluate the free radical scavenging effectiveness of various substances. From our results, the antioxidant activity of the extract (Table 3) increased with increasing concentration. Simultaneously, a similar increasing trend in antioxidant activity was recorded in the FRAP assay results, whereby the maximal concentration of the extract caused production of 3.31 FRAP units. The determination of the antioxidant potential of higher concentrations (5%, 10%, 15%, 20%, 30%) was above the limits of detection.

### 3.2. Determination of Flavonoids Using HPLC-DAD

**Figure 1:**
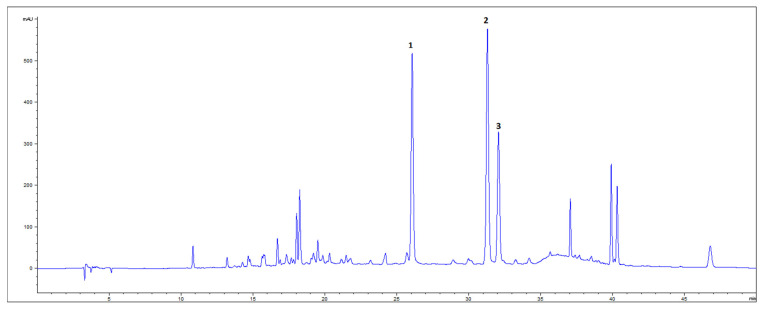
HPLC-DAD chromatogram of ethanolic extract of dry GB leaves (350 nm) after acid hydrolysis. **1**—quercetin; **2**—kaempferol; **3**—isorhamnetin.

From HPLC-DAD analyses of GBE, the presence of four flavonoid glycosides—rutin, isoquercetin, kaempferol-*3*-glucoside, and isorhamnetin-*3*-glucoside—and (+)-catechin was confirmed. After acid hydrolysis of the extract, three aglycon flavonoids—quercetin, kaempferol, and isorhamnetin—were detected (Figure 1), among which isorhamnetin was dominant in content (Table 4). The total flavonoid yield from 1 g GB DW was 0.83%, with 30% of that belonging to flavonoid aglycons and (+)-catechin detected by HPLC.

### 3.3. Vasoactivity of CAM Blood Vessels After Administration of GB

**Figure 2:**
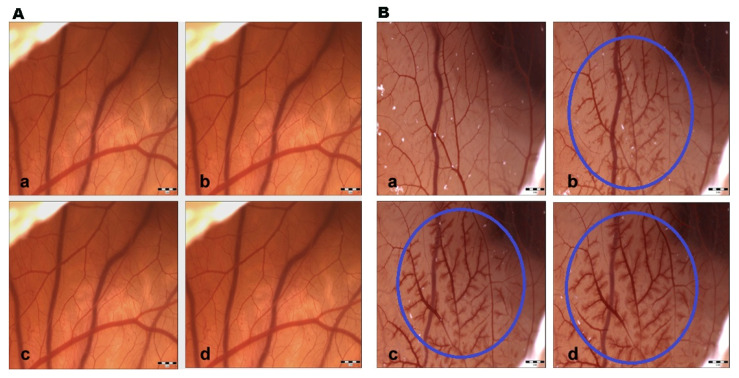
CAM blood vessel irritation potential. (**A**) Control; (**B**) 30% ethanol (positive control). a—time 0 s; b—time 30 s; c—time 120 s; d—time 240 s; circle—hyperemia with signs of hemorrhage; scale bar: 1 mm.

**Figure 3:**
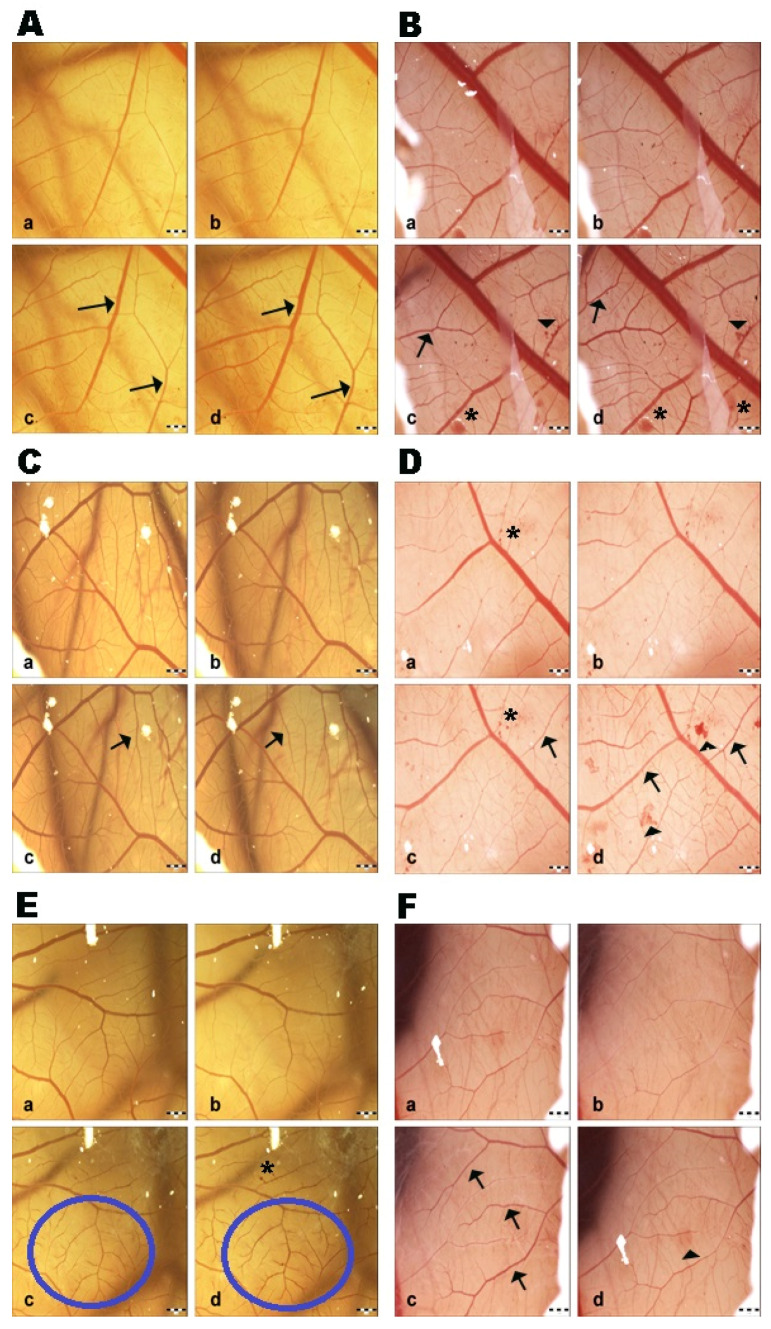
CAM blood vessel irritation potential of *Ginkgo biloba* extracts: (**A**) 1% GB extract; (**B**) 5% GB extract; (**C**) 10% GB extract; (**D**) 15% GB extract; (**E**) 20% GB extract; (**F**) 30% GB extract. a—time 0 s; b—time 30 s; c—time 120 s; d—time 240 s; arrow—hyperemia; head arrow—hemorrhage; asterisk—coagulation; circle—region of hyperemia; scale bar: 1 mm.

Saline solution did not show changes at all measured time intervals; therefore, the control group had negligible irritation potential. This fact indicates that the changes induced in the CAM vessels were made by the test substance and ethanol (positive control group) without any influence from the external environment (Figure 2). GBE exhibited hyperemia (dilatation) at all concentrations after 120 s. Subsequently, blood vessel dilatation was followed by mild hemorrhage, and coagulation also appeared at the site of vascular injury after 120 s (Figure 3). The vasoactivity effects of GBE depend not only on the time of action, but also on the concentration (Table 5). Using the 1% and 5% GBEs, vasoconstriction (disappearance of blood vessels, “ghost vessels”) was observed during the first seconds after the application of the extract. This hemostasis is not evaluated with the Luepke (1985) scoring system [19]; therefore, the changes were not evaluated. After 120 s, blood vessel dilatation occurred with moderate hemorrhage and slight coagulation. The 10% GBE showed dilatation with a moderate hemorrhage effect, mainly during the last evaluation period. Coagulation was observed only rarely. Using the 15% GB concentration, the extract manifested vascular hemorrhage and coagulation with gradual hyperemia. The cumulative score (13.75) was the highest among all tested concentrations. This shows that this concentration has the strongest irritation potential on CAM blood vessels. During the first 30 s, vasoconstriction was observed with the 20% and 30% GBEs. Subsequently, slight dilatation of blood capillaries gradually occurred. In the case of the 30% GBE, hemorrhage and coagulation were observed rarely compared to the 20% GBE. This shows that even the highest concentration of GBE appears to be safe.

The GB groups with lower concentrations (1%, 5%) had a less intense blood-vessel-damaging effect. They acted as vasodilators, meaning there was a stimulatory effect on the blood vessels.

**Figure 4:**
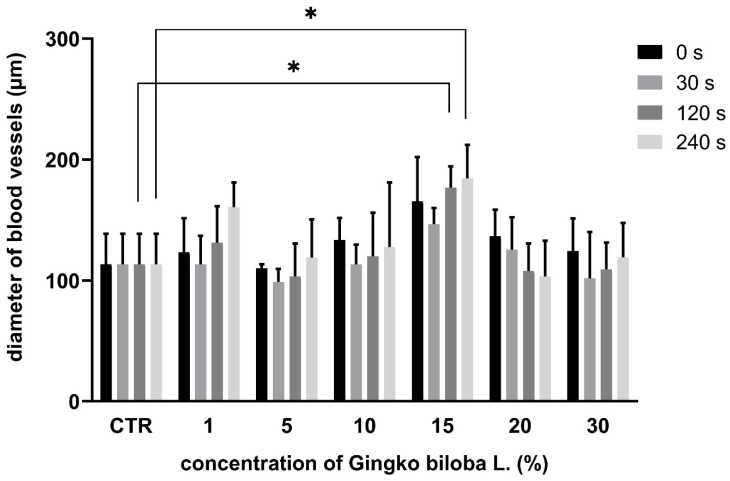
Morphometric measurement of blood vessel diameter using GB extract; * *p* < 0.005.

In the GB groups (1%, 5%, 10%, 15%), the diameter of the blood vessels was lower during the first minute after the application, which correlates with the vasoconstriction effect. But, subsequently, an increase in vessel diameter was observed. In the 20% and 30% GB groups, the diameter of the blood vessels decreased within the time of evaluation (Figure 4).

## 4. Discussion

In this work, we demonstrated the effects of natural GB extracts on the vessels of the chicken embryo chorioallantoic membrane (CAM). This extraembryonic coating forms a network of blood capillaries, which is important for the absorption of nutrients, gas transport, calcium, and the storage of waste materials. In practice, it has versatile use in the fields of angiogenesis studies, irritancy testing, evaluation of biological absorption of vasoactive drugs, transplantation, and tumor grafting research, among other areas [15].

GB is a plant that has been used in traditional medicine for many years. The presence of major secondary metabolites, such as flavonoids, biflavonoids, diterpenes, and organic acids, determines its therapeutical potential [21,22,23]. Currently, more than 30 flavonoids are confirmed in this plant. These are represented by glycosides of quercetin, kaempferol, and isorhamnetin with one, two, or three sugar units [24]. The determination of four flavonol glycosides (rutin, isoquercetin, kaempferol-*3*-glucoside, isorhamnetin-*3*-glucoside) and one flavanol ((+)-catechin) in our paper correlates with results from previous papers [25,26,27,28], in which HPLC-MS/MS and ^1^H-NMR analyses of GBE indicated the presence of quercetin, kaempferol, and isorhamnetin glycosides, simple phenols, and catechins.

Aglycone is the active component of glycoside molecules, and its release is ensured by the cleavage of glycosidic bonds by acid action via acid hydrolysis. In the case of acid hydrolysis of GBE, three main aglycons are obtained, namely quercetin, kaempferol, and isorhamnetin. In this study, their presence was confirmed too, and in addition, their amounts were comparable with the results from other studies. For example, Kobus (2009) determined kaempferol, myricetin, and isorhamnetin contents as predominant, ranging from 153 to 661 μg/g DW [29]. Similar flavonoid compositions and content ranges of related aglycons have been reported by Zhou (2017) or Ban (2020) [25,30].

The GB flavonoid composition and contents are influenced by different ecological factors such as temperature, light quality, season, and individual characteristics such as tree age and leaf extent and maturity. The leaf flavonoid content decreases with the increasing age of individuals, and flavonoid concentrations reach maximum values in the young developmental stages of trees or shoots [31]. In addition, different levels of flavonoids are recorded during the vegetative season. Lin (2020) [27] monitored the changes in the content of GB secondary metabolites from April to November and found that the highest levels of selected metabolites occurred in spring. Moreover, the authors stated the order of the major GB metabolites based on their content, on which the order of the determined flavonoids, kaempferol glycosides > quercetin glycosides > isorhamnetin, was based. This differs slightly from our results (quercetin glycosides > kaempferol glycosides > isorhamnetin); even in the case of flavonoid aglycons, we recorded a completely different order (isorhamnetin > kaempferol > quercetin). The reason for the differences may be the time of the material harvesting, the vegetative state of the plant, or the geographical location of the plant material.

The above-mentioned compounds have a high antioxidant potential due to the presence of free hydroxyl groups and multiple bonds in their molecule structures. Thanks to the dihydroxylated B-ring, quercetin and its derivatives (e.g., isorhamnetin-methoxylated derivative of quercetin) can be considered better antioxidant agents than monohydroxylated flavonoid derivatives (e.g., kaempferol) [32] or free simple phenols [33]. Because of this, quercetin glycosides are generally abundantly represented in plants, such as ginkgo. Isorhamnetin is a flavonol with four hydroxyl groups, but it offers lower antioxidant potency than quercetin precisely because of the presence of an *O*-methyl group. The reason for the increased content of free isorhamnetin can be seen in its potential to be a sort of storage form of quercetin, since the *O*-methylation of the quercetin hydroxyl group leads to a reduction in its high biological activity [34].

Many pharmacological or cosmetic preparations with antiaging, antimutagenic, anticarcinogenic, and skin-whitening properties contain compounds that originate from natural substances with antioxidant properties [35]. For commercial GB preparations intended for general use, a dry patented leaf extract (EGB 761) at a dose of 120 mg (max 240 mg) per day is recommended [36]. Approximately 25% of this extract consists of flavonoid glycosides, which is in agreement with the European Pharmacopoeia Commission. According to the European Pharmacopoeia Commission, 0.5% flavonol glycoside content is the established standard for dry GB drugs [37]. In our paper, the total content of flavonoids was in accordance with the European Pharmacopoeia. The concentrations chosen for testing the effect on CAM were lower than the recommended dose in order to take into account the sensitivity of the chosen system.

The suitability of using GBE without toxic side effects is also supported by experimental studies. Ban (2020) studied the toxic effects of flavonoid glycosides and aglycones from GBE (*Ginkgo biloba* leaf) powder. These metabolites were isolated in two ways, namely in a non-hydrolyzed (NI) and an acidolyzed isolate (AI), and were tested in a CAM model of a chicken embryo. Both isolates showed no signs of toxicity or irritant effects in the CAM model. However, the authors did not report the concentrations used [25].

The exact mechanisms of action of GBE in endothelial function are still not fully elucidated. However, a growing body of evidence suggests that its antioxidant properties play a key role. Asiwe (2023) demonstrated in his study that oral administration of GBE in rats resulted in a significant increase in the levels of vascular antioxidant enzymes such as SOD, catalase (CAT), and GSH, which contributed to the elimination of free radicals. GBE supplementation also effectively suppressed oxidative stress and inflammatory and apoptotic processes, thereby helping to restore the balance between vasoconstriction and vasodilation and promote the structural integrity of blood vessels [6]. Similarly, the results of Adebayo (2022) demonstrated that *Ginkgo biloba* has antioxidant modulatory properties in hypoxic-hypothyroid mice [38].

The main benefit of GBE is its ability to promote greater efficiency of the circulatory system by inducing vasodilation effects. It is likely that flavonoids exhibit different vasodilator actions, varying in strength. For example, myricetin and epigallocatechin gallate promote the effect of vasoconstrictor agents, which can induce an endothelium-dependent contractile response, and vice versa, flavonoids such as quercetin and kaempferol promote an endothelium-independent vasodilation mechanism [39,40]. It was previously mentioned, and our results confirm it too, that GBE contains mostly glycosides of quercetin, kaempferol, and isorhamnetin and catechin. In accordance with the content of established GB substances and vasomodulatory effects, different vasoconstrictor and vasodilator effects of GBE can be assumed, apparently dependent on the applied concentration. Our testing of the irritation effects of GBE on CAM vessels of the chicken embryo showed significant vasoactivity of the blood vessels. Although the degree and severity of effects varied between concentrations, four of the six extracts showed comparable changes. Signs of vasodilation were demonstrated, which were most pronounced at the lowest concentration of GBE. The tested extracts of GB at concentrations of 1%, 5%, and 15% had a strong irritation potential. The 10%, 20%, and 30% GBEs were characterized by a moderate irritation potential. This effect may be due to their higher concentration of biologically active metabolites, such as catechin, whereas the other flavonoids are mostly present in the glycosidically inactive forms.

The specific effect of the individual components of GBE on the CAM system and their effective concentrations should be further investigated at the molecular level of the transcription factors underlying the vasoprotective processes.

## 5. Conclusions

The objective of this study was to assess the effects of various concentrations of *Ginkgo biloba* extract on the blood vessels of the chick embryo chorioallantoic membrane (CAM), using the Luepke scoring system. Lower concentrations (1%, 5%) induced vasodilation, while higher concentrations (20%, 30%) caused vasoconstriction, accompanied by mild hyperemia and hemorrhage. HPLC analysis confirmed the presence of flavonoids, which correlated with the antioxidant activity of GBE. The selected GBE concentrations were effective and safe, without causing irreversible damage to the CAM vasculature. This study supports the use of the CAM model as an ethical tool for the preclinical testing of natural extracts in pharmaceutical research.

## Figures and Tables

**Table 1:** Scoring scheme for testing blood vessel irritation potential using CAM.

Time Taken for Manifestation of Irritation Effect	Score		
	Hyperemia	Hemorrhage	Coagulation
<0.5 min	5	7	9
0.5–2 min	3	5	7
2–5 min	1	3	5

Adapted from reference [19].

**Table 2:** Classification of irritation potential based upon cumulative score.

Cumulative Score	Irritation Potential
<1.0	Negligible
1.0–4.9	Slight
5.0–8.9	Moderate
9.0–21.0	Strong

Adapted from reference [19].

**Table 3:** Antioxidant potential of GB extract.

Ethanolic GB Extract	1 FRAP = 100 µmol/L Fe^2+^	DPPH (% Inhibition)
0.25%	2.186 ± 0.340	25.95
0.5%	2.530 ± 0.233	41.14
1.0%	2.953 ± 0.185	62.28
2.0%	3.314 ± 0.268	76.58

**Table 4:** Content of flavonoid glycosides of GB and relative aglycons after acid hydrolysis of methanolic extract (µg/g DW).

Rutin	Isoquercetin	Kaempferol-3-glucoside	Isorhamnetin-3-glucoside	(+)-Catechin
965.645 + 93.113	161.735 + 60.895	336.039 + 20.798	124.915 + 16.456	149.010 + 32.452
	**Quercetin**	**Kaempferol**	**Isorhamnetin**	
	516.696 + 22.222	769.262 + 17.825	1063.721 + 23.033	

**Table 5:** Average irritation potential of GB extract at different concentrations.

	Concentration	Hyperemia	Hemorrhage	Coagulation	Cumulative Score	Irritation Potential
Negative control	Saline solution	0	0	0	0	Negligible
Positive control	Ethanol 30%	5	0	0	5	Moderatet
Ginkgo extract	1%	3.4	3.8	2	9.2	Strong
	5%	3	5.25	2.5	10.75	Strong
	10%	2.83	3.5	0.83	7.16	Moderate
	15%	3	7	3.75	13.75	Strong
	20%	2.25	3.75	2.5	8.5	Moderate
	30%	3	1.75	1.25	6	Moderate

## Data Availability

The data presented in this study are available on request from the corresponding author. The data are not publicly available due to the fact that these data are published for the first time and authors have no problems to provide them on request.
